# CONTEXTUAL INFLUENCES ON THE MAINTENANCE OF SOCIAL INTEGRATION IN LATE LIFE

**DOI:** 10.1093/geroni/igae098.0599

**Published:** 2024-12-31

**Authors:** Oliver Huxhold

**Affiliations:** Chair

## Abstract

Being socially integrated is essential for maintaining health and well-being in late adulthood. Although life course sociology and lifespan psychology have emphasized the influence of contextual conditions on different aspects of social integration for a long time, the majority of studies on social relationships in late life have focused on differences at the individual level. To address this shortcoming, this symposium presents a number of complementary perspectives on macro- and meso-level influences on late-life social development. To this end, Webster presents an analysis that details how meso-level resources integrated into the individual’s social network enhance the individual’s likelihood of volunteering. Following this presentation, Brandt and colleagues report on societal differences across European countries in a comprehensive index of social integration and relate these differences to well-being in late adulthood. Considering short-term changes in societal conditions, Klasen and colleagues present a theoretical model that details concrete pathways by which societal crises may impact social relationships. Finally, Suanet and colleagues investigate long-term societal changes and report on cohort and socio-structural differences in age-related changes in positive and negative social exchanges. Overall, the symposium offers a comprehensive view of societal and socio-structural influences on social integration. The talks combine new theoretical perspectives with sophisticated analyses and provide new insights into contextual factors in late life that are relevant for the development of targeted social interventions.

